# Combination Therapy With Semaglutide and Dapagliflozin as an Effective Approach for the Management of Type A Insulin Resistance Syndrome: A Case Report

**DOI:** 10.3389/fendo.2022.838887

**Published:** 2022-04-14

**Authors:** José Ignacio Martínez-Montoro, José Luis Pinzón-Martín, Miguel Damas-Fuentes, Andrea Fernández-Valero, Francisco J. Tinahones

**Affiliations:** ^1^ Department of Endocrinology and Nutrition, Virgen de la Victoria University Hospital, Instituto de Investigación Biomédica de Málaga (IBIMA), Faculty of Medicine, University of Málaga, Málaga, Spain; ^2^ Centro de Investigación Biomédica en Red de la Fisiopatología de la Obesidad y Nutrición (CIBEROBN), Instituto de Salud Carlos III (ISCIII), Madrid, Spain

**Keywords:** Type A insulin resistance syndrome, glucagon-like peptide-1 receptor agonist, sodium-glucose cotransporter 2 inhibitor, diabetes, insulin resistance

## Abstract

Type A insulin resistance (IR) syndrome is a very uncommon genetic disorder affecting the insulin receptor (INSR) gene, characterized by severe IR without the presence of obesity. Patients with this condition will eventually develop diabetes, presenting a variable response to insulin-sensitizers, such as metformin and thiazolidinediones, and high doses of insulin. We report for the first time the results of the use of combination therapy with a glucagon-like peptide-1 receptor agonist and a sodium-glucose cotransporter 2 inhibitor for the treatment of diabetes in the context of type A IR syndrome.

## Introduction

Type A insulin resistance (IR) syndrome is a rare disorder due to heterozygous mutations in the insulin receptor (INSR) gene, encompassed within the group of genetic syndromes with extreme IR (which also includes leprechaunism, congenital generalized lipodystrophy, Rabson-Mendelhall syndrome and type B IR syndrome) (1, 2). It is characterized by severe IR, acanthosis nigricans and hyperandrogenism – usually with more relevant clinical features in females– in in the absence of obesity or lipodystrophy (1). Impaired glucose tolerance and diabetes mellitus may develop in some patients in this context, and insulin-sensitizers, such as metformin and thiazolidinediones, have become useful therapeutic approaches for the disease (3). However, new glucose-lowering agents may also have a role in the management of type A IR syndrome. In this case report, we communicate for the first time the use of a glucagon-like peptide-1 receptor agonist (GLP-1 RA) and a sodium-glucose cotransporter 2 inhibitor (SGLT2i) in combination therapy to improve metabolic control in a patient with Type A IR syndrome.

## Case Report

A 22-year-old Spanish man was diagnosed with diabetes at 8 years of age. He presented with polyuria, polydipsia, high blood glucose levels (319 mg/dl-17.7 mmol/L) and negative capillary ketone. He had a normal weight (body mass index-BMI 20.3 kg/m^2^, p52) and family history of type 2 diabetes mellitus (T2DM). Her mother was diagnosed with diabetes at 32 years old. She presented a normal BMI (19.5 kg/m^2^) and an excellent glycemic control (glycosylated hemoglobin -HbA1c- 5.7%; treatment: metformin 1 g/day). On the other hand, his maternal grandfather was diagnosed with diabetes at 40. He also had a normal BMI (22.8 kg/m^2^) and good glycemic control (HbA1c 7%; treatment: metformin 2 g/day and insulin glargine 42 IU/day -0.64 IU/kg/day-).

With regard to the patient, autoimmune markers, including islet cell autoantibodies, autoantibodies to GAD (GAD65) and insulin were negative, basal C-peptide was 4.19 ng/ml (reference range 0.81-4.5 ng/ml) and there were no signs of acanthosis nigricans. GCK-MODY and HNF1A-MODY genetic tests were also negative. Insulin therapy was started, but the patient lost follow-up and abandoned treatment. 5 years later, the patient retook follow-up visits. He did not refer previous history of relevant diseases apart from diabetes, diabetes-related complications or surgical interventions and was not taking any medication. The patient continued presenting a normal BMI (21.23 kg/m^2^), with a HbA1c of 7.7% (61mmol/mol) and acanthosis nigricans. Basal insulinemia was 134.9 µU/ml (reference range 3-25 µU/ml), C-peptide 5.24 ng/ml and the homeostasis model assessment of IR (HOMA-IR) was elevated (23.6). Insulin immunoassay during oral glucose tolerance test showed values over 1000 µU/ml and INSR genetic test revealed an heterozygous mutation in exon 20 (p.Asp1177Glu, C.35441 C>G) (a newly- identified mutation in the beta-chain region of the INSR gene, also present in patient’s mother and grandfather). Importantly, previous functional studies have identified mutations in this region associated with IR phenotype (1). Therefore, the diagnosis of type A IR syndrome was established. Treatment with metformin 850 mg/day was initiated, achieving an adequate metabolic control (HbA1c 6.6%- 49 mmol/mol), and fasting insulinemia decreased to 112.6 µU/ml.

After two years of glycemic stability, a slight worsening of metabolic control (HbA1c 7%- 53 mmol/mol) led to an increase of metformin dose (1275 mg/day) and the addition of sitagliptin (50 mg/day). However, the patient suffered a progressive deterioration of glycemic control (HbA1c levels between 8.5%- 69 mmol/mol and 10%-86 mmol/mol and marked postprandial hyperglycemia). Metformin and sitagliptin daily doses were increased (2000 mg and 100 mg, respectively) and glimepiride 4 mg/day and pioglitazone 15 mg/day were added, although the latter was suspended two months after its initiation because of significant peripheral edema. In addition, basal-bolus insulin therapy was started (insulin glargine and lispro), reaching 156 IU/day (23% basal and 77% bolus, total insulin dose 2.23 IU/kg/day). The patient also received nutritional counselling (2400 kcal/day, 50% carbohydrate, 20% protein, 30% fat) and physical activity recommendations (moderate-intensity aerobic exercise 45 min/day). However, the poor metabolic control persisted. During this period of time, the patient had maintained a normal BMI (22.2-22.9 kg/m^2^).

**Figure 1:**
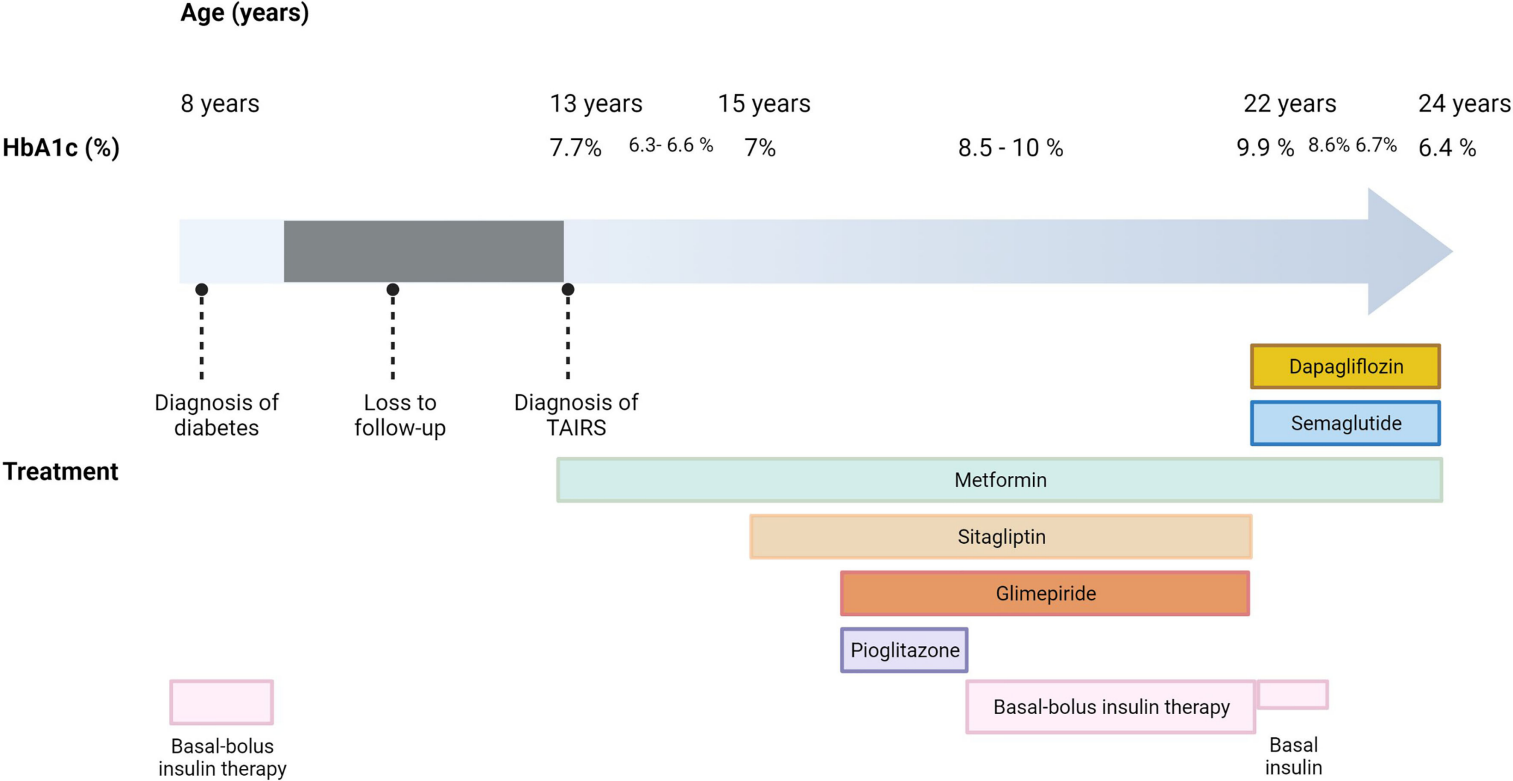
Clinical course of glycemic control and treatment timeline in the reported case of type A insulin resistance syndrome. HbA1c, glycosylated hemoglobin; TAIRS, type A insulin resistance syndrome.

In order to improve metabolic control, dual therapy with a GLP-1 RA and a SGLT2i was proposed, given the reported benefits of this combination (4–6). Therefore, subcutaneous semaglutide 0.25 mg/week and dapagliflozin 10 mg/day were prescribed, whereas sitagliptin 100 mg/day and glimepiride 4 mg/day were stopped. The patient continued taking metformin. Also, he was recommended to decrease total daily insulin dose to avoid hypoglycemia. Before these modifications, the patient had a BMI of 24.8 kg/m^2^ and a HbA1c of 9.9% (85 mmol/mol). One month after the therapeutic change, the patient lost 4 kg and presented a HbA1c of 8.6% (70 mmol/mol). Besides, he was not using prandial insulin. Six months after treatment adjustment (semaglutide was increased to 0.5 mg/week after 4 weeks), he lost 10 kg and achieved a good metabolic control (HbA1c 6.7%- 50 mmol/mol). Moreover, he had stopped insulin therapy and presented a decrease of basal insulinemia (47 µU/ml). Two years after the initiation of combination therapy with dapagliflozin and semaglutide, the patient continues with an excellent glycemic control (HbA1c 6.4%- 46 mmol/mol). Clinical course of glycemic control and treatment timeline are summarized in **Figure 1**.

## Discussion

The management of patients with diabetes in the context of type A IR syndrome should be focused on achieving an optimal metabolic control to avoid long-term complications associated with this disease. In this regard, metformin and pioglitazone have been used as therapeutic strategies to improve insulin sensitivity (3). Treatment with recombinant insulin growth factor (IGF)-1 or IGF-1/IGF binding protein-3 may also be effective, but this therapy is expensive and there are no available studies assessing its long-term benefit (7). Thus, a number of patients will require high doses of exogenous insulin, with heterogeneous response.

The therapeutic rationale for starting combination therapy with semaglutide and dapagliflozin in this patient was based on several premises. First of all, these are therapeutic decisions in successive steps depending on the success or failure of the previous one. In this regard, previous experience with “multistep” therapy was described by Moreira et al. in a case of extreme IR (Rabson-Mendenhall syndrome) (8). Previous studies have also reported promising results with regard to several SGLT2is, such as ipragliflozin and dapaliflozin, in the treatment of diabetes in patients with type A IR syndrome (9, 10). Accordingly, Nagashima et al. showed that the administration of ipragliflozin led to significant reductions in HbA1c (10% to 7.2%, maintained at approximately 8% for more than 3 years) in a patient with type A IR syndrome treated with metformin and high doses of insulin (9). Similarly, Hamaguchi and colleagues reported that dapagliflozin reduced HbA1c from 7.5% to 6.5% and decreased total daily dose of insulin in a case of severe IR due to a PIK3R1 mutation treated with metformin and insulin (10). Therefore, given the poor glycemic control observed in our patient (HbA1c 9.9%) despite the use of multiple oral glucose-lowering agents and high doses of insulin, we considered that the addition of a single therapy to his medication would not be enough to decrease HbA1c ≤ 6.5% and/or significantly reduce total daily dose of insulin. Thus, we started combination therapy with a GLP-1 RA and a SGLT2i. Indeed, in a randomized controlled trial including subjects with T2DM inadequately controlled by metformin, co-initiation of exenatide and dapagliflozin significantly reduced HbA1c compared with each of these drugs alone (4). Moreover, a recent meta-analysis of 8 randomized controlled trials confirmed the efficacy and safety of this combination compared with monotherapy (5). However, these studies were conducted in subjects with T2DM, and there are no data concerning to GLP-1 RA and SGLT2i co-initiation in individuals with type A IR syndrome.

Combination therapy with a GLP-1 RA and a SGLT2i has been reported to involve additional benefits in the management of T2DM compared with monotherapy through different mechanisms (11). In this regard, when used in combination, these pharmacological agents complement each other to correct several components of the Ominous Octet described by DeFronzo (11). On the one hand, GLP-1 RAs enhance insulin release and inhibit glucagon secretion, leading to a reduction in hepatic glucose production; they improve gastrointestinal incretin defect; they inhibit appetite and induce weight loss and, indirectly, weight loss enhances muscle and hepatic insulin sensitivity (11). On the other hand, SGLT2is suppress glucose reabsorption in the proximal tubule, which results in glucosuria, ameliorating glucotoxicity and improving beta cell function (11). Interestingly, beyond these mechanisms, SGLT2is may improve metabolic control in type A IR syndrome *via* an improvement in insulin sensitivity (12), whereas GLP-1 RAs may also have similar effects, regardless of the presence of overweight/obesity (13). It is important to note that, as far as we know, there are no available studies/case reports assessing the role of GLP-1 RAs in type A IR syndrome. In line with our results, the use of GLP-1 RAs or the combination therapy with GLP-1RAs and SGLT2is may become an attractive option for the treatment of type A IR syndrome. However, we cannot predict how long this treatment will be effective. Although a short-term successful response has been observed, deterioration of glycemic control is likely over time. Since, to our knowledge, this is the first case report that describes the effectiveness of dual therapy with a GLP1-RA and a SGLT2i in type A IR syndrome, further research is needed to assess the role of these glucose-lowering agents in the treatment of this condition.

## Data Availability Statement

The original contributions presented in the study are included in the article/supplementary material. Further inquiries can be directed to the corresponding authors.

## Ethics Statement

This study was conducted ethically in accordance with the World Medical Association Declaration of Helsinki. Informed consent was obtained from the patient for the publication of the details of their medical case and the accompanying images.

## Author Contributions

JM-M: conceptualization, data curation writing-original draft and writing-review & editing. JP-M: conceptualization, data curation and writing-review & editing. MD-F: data curation and writing-review & editing. AF-V: writing-review & editing. FT: supervision and writing-review & editing. All authors participated in drafting the article or revising it critically for important intellectual content and gave their final approval of the version to be submitted.

## Funding

This study was supported by “Centros de Investigación Biomédica en Red” (CIBER) of “Instituto de Salud Carlos III” (ISCIII) (CB06/03/0018) and research grants from the ISCIII (PI18/01160), and co-financed by the European Regional Development Fund (ERDF).

## Conflict of Interest

The authors declare that the research was conducted in the absence of any commercial or financial relationships that could be construed as a potential conflict of interest.

## Publisher’s Note

All claims expressed in this article are solely those of the authors and do not necessarily represent those of their affiliated organizations, or those of the publisher, the editors and the reviewers. Any product that may be evaluated in this article, or claim that may be made by its manufacturer, is not guaranteed or endorsed by the publisher.

## References

[1] Semple RK Savage DB Cochran EK Gorden P O’Rahilly S. Genetic Syndromes of Severe Insulin Resistance. Endocr Rev (2011) 32(4):498–514. doi: 10.1210/er.2010-0020 21536711

[2] Hattori Y Satoh H Namatame T Hattori S Kasai K. A Patient With Extreme Insulin Resistance Syndrome Treated With Pioglitazone. Diabetes Care (2006) 29(10):2328–9. doi: 10.2337/dc05-1249 17003320

[3] Semple RK Williams RM Dunger DB. What is the Best Management Strategy for Patients With Severe Insulin Resistance? Clin Endocrinol (Oxf) (2010) 73(3):286–90. doi: 10.1111/j.1365-2265.2010.03810.x 20455892

[4] Frías JP Guja C Hardy E Ahmed A Dong F Öhman P. Exenatide Once Weekly Plus Dapagliflozin Once Daily Versus Exenatide or Dapagliflozin Alone in Patients With Type 2 Diabetes Inadequately Controlled With Metformin Monotherapy (DURATION-8): A 28 Week, Multicentre, Double-Blind, Phase 3, Randomised Control. Lancet Diabetes Endocrinol (2016) 4(12):1004–16. doi: 10.1016/S2213-8587(16)30267-4 27651331

[5] Li C Luo J Jiang M Wang K. The Efficacy and Safety of the Combination Therapy With GLP-1 Receptor Agonists and SGLT-2 Inhibitors in Type 2 Diabetes Mellitus: A Systematic Review and Meta-Analysis. Front Pharmacol (2022) 13:838277. doi: 10.3389/fphar.2022.838277 PMC8854770 35185588

[6] Lajara R. Combination Therapy With SGLT-2 Inhibitors and GLP-1 Receptor Agonists as Complementary Agents That Address Multi-Organ Defects in Type 2 Diabetes. Postgrad Med (2019) 131(8):555–65. doi: 10.1080/00325481.2019.1670017 31580737

[7] Regan FM Williams RM McDonald A Umpleby AM Acerini CL O’Rahilly S. Treatment With Recombinant Human Insulin-Like Growth Factor (rhIGF)-I/rhIGF Binding Protein-3 Complex Improves Metabolic Control in Subjects With Severe Insulin Resistance. J Clin Endocrinol Metab (2010) 95(5):2113–22. doi: 10.1210/jc.2009-2088 20233784

[8] Moreira RO Zagury RL Nascimento TS Zagury L. Multidrug Therapy in a Patient With Rabson–Mendenhall Syndrome. Diabetologia (2010) 53(11):2454–5. doi: 10.1007/s00125-010-1879-5 20711714

[9] Nagashima S Wakabayashi T Saito N Takahashi M Okada K Ebihara K. Long-Term Efficacy of the Sodium–Glucose Cotransporter 2 Inhibitor, Ipragliflozin, in a Case of Type A Insulin Resistance Syndrome. J Diabetes Investig (2020) 11(5):1363–5. doi: 10.1111/jdi.13241 PMC7477530 32100949

[10] Hamaguchi T Hirota Y Takeuchi T Nakagawa Y Matsuoka A Matsumoto M. Treatment of a Case of Severe Insulin Resistance as a Result of a PIK3R1 Mutation With a Sodium-Glucose Cotransporter 2 Inhibitor. J Diabetes Investig (2018) 9(5):1224–7. doi: 10.1111/jdi.12825 PMC6123033 29476696

[11] DeFronzo RA. Combination Therapy With GLP-1 Receptor Agonist and SGLT2 Inhibitor. Diabetes Obes Metab (2017) 19(10):1353–62. doi: 10.1111/dom.12982 PMC5643008 28432726

[12] Yaribeygi H Sathyapalan T Maleki M Jamialahmadi T Sahebkar A. Molecular Mechanisms by Which SGLT2 Inhibitors Can Induce Insulin Sensitivity in Diabetic Milieu: A Mechanistic Review. Life Sci (2020) 240:117090. doi: 10.1016/j.lfs.2019.117090 31765648

[13] Jiang Y Wang Z Ma B Fan L Yi N Lu B. GLP-1 Improves Adipocyte Insulin Sensitivity Following Induction of Endoplasmic Reticulum Stress. Front Pharmacol (2018) 9:1168. doi: 10.3389/fphar.2018.01168 30459598 PMC6232689

